# Altered drug susceptibility during host adaptation of a *Plasmodium falciparum* strain in a non-human primate model

**DOI:** 10.1038/srep21216

**Published:** 2016-02-16

**Authors:** Nicanor Obaldía III, Geoffrey S. Dow, Lucia Gerena, Dennis Kyle, William Otero, Pierre-Yves Mantel, Nicholas Baro, Rachel Daniels, Angana Mukherjee, Lauren M. Childs, Caroline Buckee, Manoj T. Duraisingh, Sarah K. Volkman, Dyann F. Wirth, Matthias Marti

**Affiliations:** 1 Department of Immunology and Infectious Diseases, Harvard | T.H. Chan School of Public Health, Boston, MA, United States; 2 Walter Reed Army Institute of Research, Silver Springs, MD, United States; 3 Department of Global Health, University of South Florida, Tampa, FL, United States; 4 Center for the Evaluation of Antimalarial Drugs and Vaccines, Tropical Medicine Research/Instituto Conmemorativo Gorgas de Estudios de la Salud, Panamá City, Republic of Panama; 5 Center for Communicable Disease Dynamics and Harvard | T.H. Chan School of Public Health, Boston, MA, United States; 6 Department of Epidemiology, Harvard | T.H. Chan School of Public Health, Boston, MA, United States; 7 The Broad Institute of MIT and Harvard, Cambridge, MA, United States; 8 School of Nursing and Health Sciences, Simmons College, Boston, MA United States

## Abstract

Infections with *Plasmodium falciparum,* the most pathogenic of the *Plasmodium* species affecting man, have been reduced in part due to artemisinin-based combination therapies. However, artemisinin resistant parasites have recently emerged in South-East Asia. Novel intervention strategies are therefore urgently needed to maintain the current momentum for control and elimination of this disease. In the present study we characterize the phenotypic and genetic properties of the multi drug resistant (MDR) *P. falciparum* Thai C2A parasite strain in the non-human *Aotus* primate model, and across multiple passages. *Aotus* infections with C2A failed to clear upon oral artesunate and mefloquine treatment alone or in combination, and *ex vivo* drug assays demonstrated reduction in drug susceptibility profiles in later *Aotus* passages. Further analysis revealed mutations in the *pfcrt* and *pfdhfr* loci and increased parasite multiplication rate (PMR) across passages, despite elevated *pfmdr1* copy number. Altogether our experiments suggest alterations in parasite population structure and increased fitness during *Aotus* adaptation. We also present data of early treatment failures with an oral artemisinin combination therapy in a pre-artemisinin resistant *P. falciparum* Thai isolate in this animal model.

Antimalarial drug resistance is one of the greatest threats to the current malaria eradication agenda^1^. Oral artemisinin-based combination therapy (ACT) is the standard of care for uncomplicated malaria, while parenteral intravenous (i.v.) treatment is used in severe cases. Artemisinin (QHS) is the fast acting component of ACTs that accelerates clearing of young ring stage parasites by the spleen^2^. In 2009 decreased susceptibility to AS was first observed in western Cambodia^3^, and has since been detected in the rest of Southeast Asia, including recent reports of clinical treatment failure of ACTs such as artesunate/mefloquine (AS/MQ) or artemether/lumefantrine (AL or Coartem)^4^,^5^. *In vitro* selection experiments followed by genome-wide association studies have meanwhile identified mutations in the *PF3D7_1343700* (*PF13_0238*) genomic locus encoding a Kelch protein that are strongly correlated with slow clearing parasites^6^. Such mutations appear to occur in the context of a specific genetic background in Southeast Asia but so far have not been detected in Africa^7^,^8^.

The TM90C2A (C2A) parasite is a multidrug resistant *P. falciparum* strain originally isolated from a patient in Thailand in 1992, prior to the observation of altered susceptibility to QHS in Southeast Asia^9^. Initial genotyping demonstrated presence of quadruple-mutations in the *dhfr-thymidylate synthase* gene^10^ and increased *pfmdr1* copy number^11^. This combination of mutations and *pfmdr1* copy number variation (CNV) has been associated in field isolates with higher *in vitro* inhibitory concentrations to MQ, quinine (QN), halofantrine and QHS, and with failure of MQ monotherapy and AS/MQ combination therapy at the Thai-Cambodian border^12^,^13^. Notably, AS/MQ was deployed in 1994 in Thailand and adopted as standard treatment by the Thai health authorities in 2005. However, QHS monotherapy had been used in western Cambodia since the late 1970’s and MQ was introduced in Thailand in 1984^3^.

Recently, we reported the adaptation of the Thai C2A clone to splenectomized *Aotus l. lemurinus* monkeys (Panamanian Owl monkey). *Plasmodium* infection in *Aotus* was first reported in Panama at the Gorgas Memorial Laboratory in 1966 and has since been established as a major non-human primate model to host-parasite-vector interactions, immunology and pathophysiology and evaluation of drugs and vaccines in *P. falciparum* and *P. vivax*^14^. During the adaptation process of passaging the parasite in *Aotus*, we observed decreasing susceptibility of the parasites to oral MQ or i.v. AS alone or in combination^15^. To determine the basis for the altered drug sensitivity phenotype during *Aotus* adaptation, we tested the antimalarial drug efficacy to MQ and artemisinin derivatives *in vivo* and *in vitro* in C2A, and correlated these parameters with growth rates, genotypic changes and mutations in antimalarial drug resistance genes.

## Results

### Artesunate treatment failure with the C2A *P. falciparum* strain in the *Aotus* model

**Figure 1:**
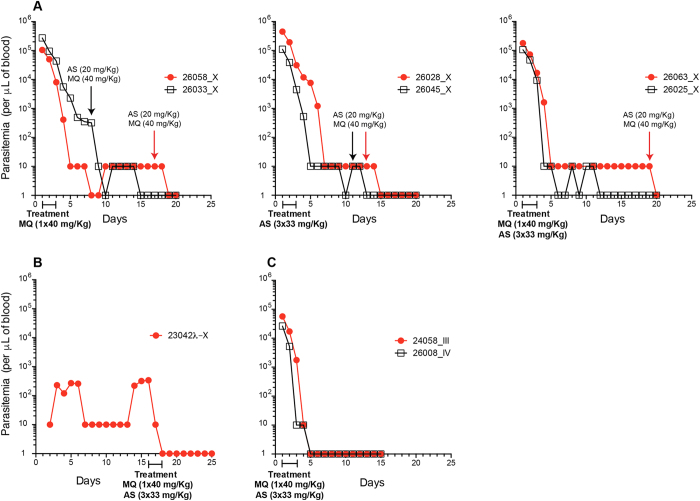
Antimalarial drug responses of *Plasmodium falciparum* C2A infected *Aotus* monkeys. (**A**) Parasitemia response plots of *Aotus* monkeys infected with the *P. falciparum* C2A clone passage X. Animals were inoculated with 5 × 10^6^ infected red blood cells from a donor monkey (MN26006, passage IX) and treated when parasitemia reached >10^5^ parasites × μL of blood. Panel (**A**) shows group 1 animals treated with MQ at 40 mg/Kg orally once, group 2 animals treated with AS at 33 mg/Kg orally × three days and group 3 animals treated with MQ and AS at 33 mg/Kg × three days and MQ at 40 mg/Kg orally once. Panel (**B**) shows a spleen intact control treated with AS at 33 mg/Kg × three days +MQ at 40 mg/Kg orally once on day 16 of infection. Panel (**C**) shows parasitemia responses of MN24058 and MN26008 inoculated with PfC2A passages III and IV respectively and treated with AS at 33 mg/Kg × three days i.v. +MQ at 40 mg/Kg orally once.

We previously demonstrated treatment failures upon AS treatment with the multidrug resistant C2A in the *Aotus* monkey model^15^. Specifically, we observed recrudescence in monkey passage VII and suppression in passage VIII, in each case after treatment with AS at 20 mg/Kg i.v. for three days^15^. To systematically investigate this drug susceptibility phenotype we performed a controlled experiment in *Aotus* and followed parasitemia upon oral AS treatment alone, or in combination with MQ. Malaria naive and splenectomized male and female *Aotus lemurinus lemurinus* monkeys were inoculated with 5 × 10^6^ parasites of the C2A clone on its IX serial passage from a donor *Aotus* and further divided into three oral treatment groups of two monkeys each (Fig. 1, Table 1): one group was treated with a single dose of 40 mg/Kg of oral MQ; a second group was treated with daily doses of 33 mg/Kg of oral AS for three days; and a third group was treated with a combination of a single dose of oral MQ and three daily doses of oral AS. As a control a spleen intact animal was also infected and treated with the same dose regimen as the third group. Parasitemia was patent in all animals between days 1–2 post infection (PI), reaching peak parasitemia of >100,000 parasites/μL in groups 1–3 by day 9–11 PI. In contrast, a peak parasitemia of 340 parasites/μL on day 16 PI was reached in the spleen intact animal. On the first day >100,000 parasites/μL were reached treatment was initiated (Day 0 in Fig. 1).

In each of the three treatment regimes, the animals remained parasitemic with 10–100 parasites/μL for >10 days (Fig. 1A, Table 1 and S3). In the spleen intact animal, parasitemia persisted at low levels until treatment on day 16 PI and was cleared by day 3 of treatment (Fig. 1B). In contrast, when splenectomized animals were infected with parasites from *Aotus* passages III and IV and treated i.v. with AS at 33 mg/Kg for 3 days and MQ at 40 mg/Kg once at peak parasitemia (<100,000 parasites/μL), parasites were cleared on average on day 5 following initiation of treatment (Fig. 1C, Table S3). Similar clearance times are found in human cohorts: in one study 25% of patients with parasitemia >4% (>100,000 parasites/μL) were positive beyond day 3 of treatment, but only 5% with parasitemia <4%^16^. Notably, the i.v. AS dosage used in our study is about eight-fold higher than what is commonly given to humans for oral treatment of uncomplicated^17^,^18^ or severe malaria (33 mg/Kg *versus* 4 mg/kg)^19^. Converting the *Aotus* animal dose of AS used in this study (33 mg/Kg) to a human equivalent dose based on body surface area^20^ would result in ~11 mg/Kg, approximately three times the human standard dose of 4 mg/Kg. Together these experiments demonstrate that infections with the MDR C2A isolate in the *Aotus*/*P. falciparum* non-human primate model show characteristics of MQ resistance and reduced AS susceptibility, including parasite suppression upon i.v. or oral AS at 33 mg/Kg × three days, or in combination with MQ at 40 mg/Kg once (Fig. 1).

### C2A shows altered drug susceptibility profiles *ex vivo*

**Figure 2:**
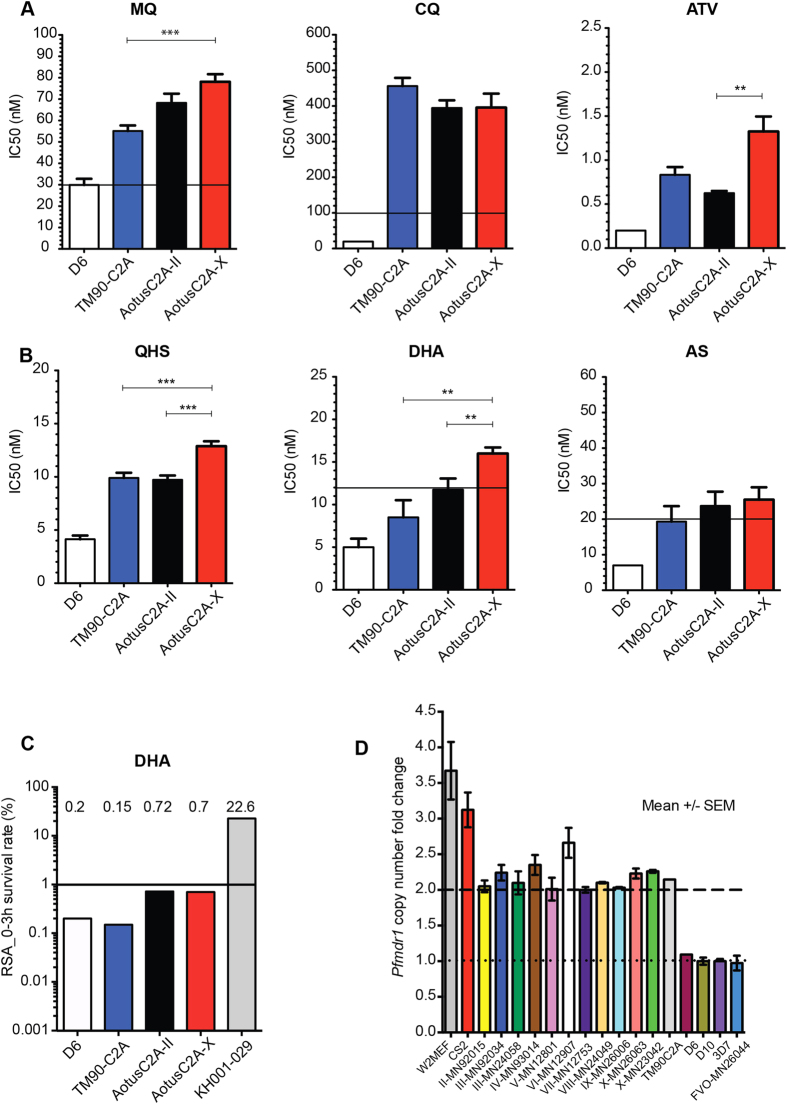
*Ex vivo*/*in vitro* antimalarial drug susceptibility and Pfmdr1 copy number variation of *Pf*C2A across Aotus passages. (**A**,**B**) IC_50_ antimalarial drug nM concentration bar plots of reference strains D6 (white bar), *in vitro* culture adapted TM90C2A (blue bar) and *pf*C2A *Aotus* adapted passage levels II (black bar) and X (red bar). Drug IC50 nM resistance threshold (black line). Mean ± sample SEM (see also Table S1). (**C**) DHA drug susceptibility based on RSA. Survival percentage = DHA treated parasites/DMSO treated parasites × 100. Red bar represents survival of artemisinin resistant positive control strain KH001-029, D6 is included as negative reference strain. Significant survival threshold (black line) is set at 1% as in Witkowski *et al.*^24^. (**D**) Passage level *pfmdr1* copy number fold change of a *Plasmodium falciparum* C2A clone during adaption to *Aotus* monkeys. *P. falciparum* strains with two *pfmdr1* gene copies are considered resistant to MQ. Mean ± sample SEM. Dashed line indicates threshold for *pfmdr1* CN level indicative of MQ resistance. Dotted line indicates threshold for *pfmdr1* CN level indicative of MQ sensitivity. MQ = Mefloquine; CQ = Chloroquine; ATV = Atovaquone; AS = Artesunate; QHS = Artemisinin; DHA = Dihydroartemisinin. *p* = Mann-Whitney U significance t test unpaired samples. ***p < 0.005; **p < 0.05.

To further investigate the drug resistance profile of the C2A strain we performed *ex vivo* assays of C2A from several *Aotus* passages with a series of antimalarial compounds. Specifically, we determined the *ex vivo* IC_50_ values for *Aotus* passages II and X and the original *in vitro*-adapted TM90C2A isolate against MQ, CQ, atovaquone (ATV), QHA, DHA and AS (Fig. 2). As a control we also measured the IC_50_ of the sensitive D6 strain (CQ and MQ susceptible). These experiments confirmed that TM90C2A is indeed resistant to MQ, CQ and ATV (Fig. 2A), as observed in our previous experiments in the *Aotus* model^15^. MQ IC_50s_ are >30 nM, a concentration that has been associated with treatment failures in infected patients^21^,^22^. Interestingly we also observed reduced susceptibility with C2A to the three different artemisinin derivatives, QHA, DHA and AS, and compared to D6 (Fig. 2B). IC_50_ concentrations were above the previously defined thresholds of 12 nM^21^ for DHA (C2A passage X), and 20 nM^21^,^23^ for AS (C2A passages II and X). Interestingly, parasites showed a significant decrease in susceptibility against MQ, QHS and DHA between *in vitro*-adapted TM90C2A and C2A passages II and X. In contrast, CQ IC_50_ was not significantly different across C2A passages. To further evaluate possible reduction in artemisinin susceptibility across C2A passages, we performed the recently established ring stage assay (RSA)^24^. This experiment indeed demonstrated some reduced susceptibility for *ex vivo* C2A passages II and X compared to the *in vitro*-adapted original TM90C2A isolate and the D6 control (Fig. 2C), although still below the defined threshold of 1% ring stage survival for resistance^24^. Altogether these data demonstrated phenotypic changes in C2A across *Aotus* passages such as altered susceptibility to QHS, DHA, and MQ. Interestingly the *in vitro*-adapted TM90C2A did not show any signs of altered artemisinin susceptibility while being equally resistant to MQ and CQ compared to the *Aotus* passages.

### Genetic analysis of C2A during *Aotus* passages and *in vitro* adaptation

To track possible changes in the genetic composition of C2A across *Aotus* passages and during *in vitro* adaptation we first analyzed copy number variation (CNV) of *pfmdr1* (Fig. 2D). These experiments demonstrated variation in *pfmdr1* copy number between 2 and 2.5 across all passages, and copy number of 2 in the *in vitro*-adapted line, suggesting presence of mixed genotypes across *Aotus* passages.

**Figure 3:**
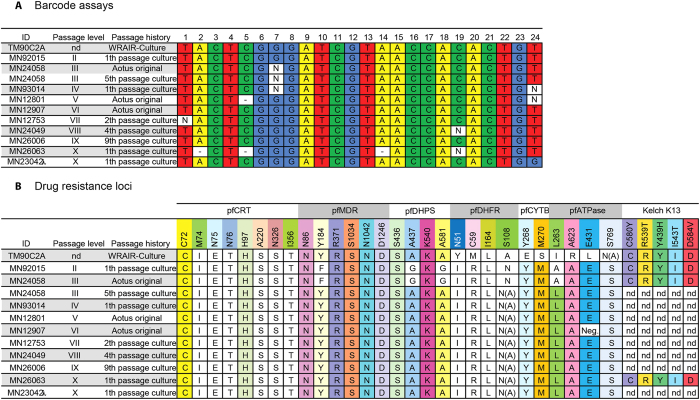
Molecular barcode and antimalarial drug resistance loci across C2A passages. (**A**) *Plasmodium falciparum* barcode. (**B**) Drug resistance genotyping profiles. nd = not determined; λ = spleen intact.

Given the *pfmdr1* CNV data and the observed drug resistant profiles with C2A, we performed detailed genetic analysis of C2A across passages and with the *in vitro*-adapted TM90C2A. To determine whether C2A represented a single genotype across passages, despite the observed *pfmdr1* CNV, we performed genotyping using a molecular barcode assay that assesses the parasite genotype based on a combination of 24 neutral loci across the 14 *P. falciparum* chromosomes^25^. Genotyping by molecular barcode assay demonstrated that C2A parasites across all passages from TM90C2A to passage X show identical or very closely related major haplotypes (Fig. 3A). Genetic analysis of drug resistance loci revealed resistance mutations across all passages including a mutation in the *in vitro*-adapted TM90C2A within the *pfcrt* (M74I, N75E, N76T, A220S, N326S, I356T) and *pfdhfr* (N51I, C59R, I164L, S108N) loci (Fig. 3B). No mutations were found in the *Kelch K13* locus across TM90C2A and passage II, III and X. However, compared to the 3D7 reference sequence we found a tandem asparagine insert (NN) after codon 142 in the propeller domain in passages II, III and X as well as in the *in vitro*-adapted TM90C2A. This mutation has been observed in other parasites and is not related to an artemisinin resistance phenotype^26^. Interestingly specific mutations in *pfmdr1* (Y184F), *pfdhps* (A437G, A581G) and *pfATPase* (L263A) were only found in passages II and III (Fig. 3B). Similarly distinct sets of mutations were found in the *in vitro*-adapted TM90C2A in *pfdhfr* (N51Y, C59M) and *pfATPase* (L263I, A623R, I431L, S769A/N), and these parasites also harbor unique mutations in *pfcytb* (Y268E, M270S). Given the molecular barcode data these observations suggest that the original C2A strain was a mixture of closely related parasite genotypes from which at least two were selected during subsequent *Aotus* passages (represented by passages II/III and passages IV-X, respectively) and another genotype was selected during *in vitro* adaptation (TM90C2A).

### Serial passaging of C2A in *Aotus* selects for increased growth phenotype

**Figure 4:**
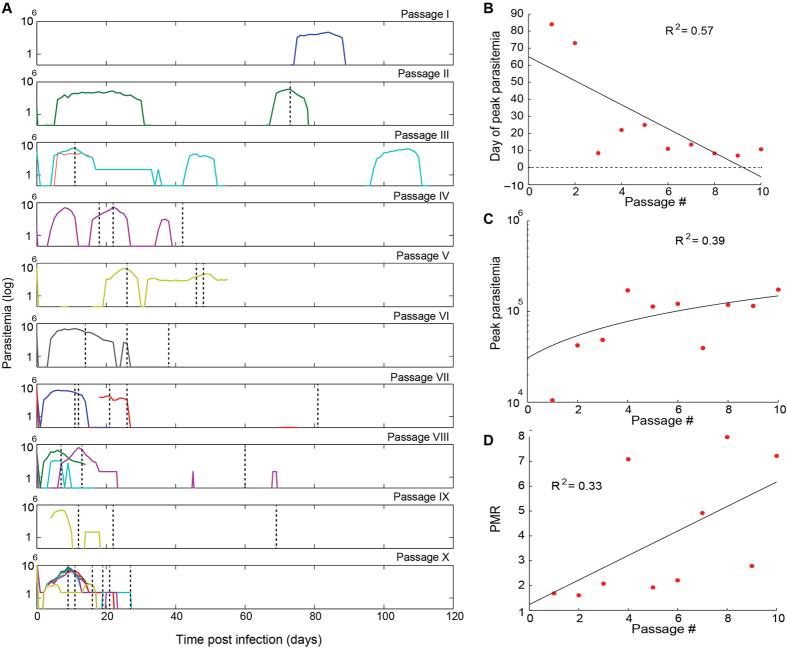
*Plasmodium falciparum* C2A growth across *Aotus* passages. (**A**) Parasitemia plots from individual monkeys (colored lines) with time of treatment (black dotted lines) across each passage. Parasitemia was followed for up to 100 days post inoculation and frequently fell below detectability (disappearance of colored lines). (**B**) Day of peak parasitemia trend. The day of peak parasitemia is the time since inoculation when peak parasitemia was achieved, averaged across monkeys for each passage. (**C**) Peak parasitemia trend. The value of peak parasitemia is the maximum parasitemia reached over the course of infection, averaged across monkeys for each passage. (**D**) Parasite multiplication rate (PMR). The PMR is the median of parasitemia increases across 48-hr periods over the course of an infection, averaged across monkeys for each passage. The data is modeled with linear regression.

As in other biological systems *P. falciparum* adaptation (*in vitro* and in animals) selects for the genotypes with maximal growth in the absence of any other selection (i.e., drug pressure)^27^,^28^,^29^. Increased growth rates typically result in decreased drug susceptibility profiles as demonstrated in the rodent malaria model^30^. We therefore wanted to quantify the altered parasite growth rates across *Aotus* passages, as a possible contributor to the observed changes in drug susceptibility. We analyzed growth rates during *Aotus* adaptation based on the recorded parasitemia from each of the 10 passage experiments (Fig. 4A), considering the time (Fig. 4B) and peak parasitemia (Fig. 4C). Specifically we estimated parasite multiplication rate (PMR, Fig. 4D) from the median of all increases in parasitemia at 48-hour intervals averaged across monkeys in the same passage. These data demonstrated that parasite growth increased significantly from a PMR just above 1 in passage I to PMR of above 7 in passage X. Interestingly increased growth rates were not linked to genomic deletions on chromosomes 2 and 9 (Fig. S1) that occur frequently during *in vitro* parasite adaptation^31^ and have been observed phenotypically in a splenectomized monkey model^32^.

## Discussion

In a previous study we observed MQ and AS treatment failures with the Thai multi-drug resistant *Pf*C2A in *Aotus* monkeys^15^. Here we systematically investigate the antimalarial drug responses of this isolate and its changes during host adaptation.

Artemisinin resistance in humans is defined as reduced parasite clearance rate or persistence of microscopically detectable parasites on the third day of ACT therapy^33^. We observed suppression and persistence of microscopically detectable parasites beyond the third day of oral AS alone or in combination with MQ, indicating resistance of the *Aotus*-adapted PfC2A isolate based on the above definition. The observed phenotype is unlikely due to limited AS and/or MQ bioavailability by the oral delivery route as our previous experiments in *Aotus* with the FVO *P. falciparum* strain have efficiently cleared parasites at day 4 when AS was administered i.v. or orally at 8 mg/Kg for three days^34^. Similarly MQ treatment as a single dose of 20 mg/Kg cleared parasites on day 5 following initiation of treatment in *Aotus* infected with FVO^35^. However, we cannot exclude that an inoculum effect^36^ may have contributed to the observed relative lower efficiency of the oral route at high parasitemia densities (≥100 × 10^3^/μL) compared to the i.v. route at low parasitemia (<1.0 × 10^3^/μL). Finally, splenectomy may have resulted in reduced efficiency of artemisinin-mediated parasite clearance as discussed previously^35^.

*In vitro* and *ex vivo* experiments demonstrated reduced DHA susceptibility at later *Aotus* passages, although remaining below the standard resistance threshold of 1% ring stage survival. Genotyping experiments demonstrated presence of wild type *Kelch K13* locus across all C2A passages, therefore ruling out involvement of this major resistance locus in the observed *in vivo* and *in vitro* or *ex vivo* drug susceptibility phenotypes. We also demonstrated treatment failure upon MQ treatment in *Aotus* and MQ drug resistant phenotype *in vitro* and *ex vivo. Pfmdr1* copy number fluctuated between 2 to 2.5 during *Aotus* adaptation while MQ susceptibility decreased at later passages. These observations contrast with *in vitro* studies where *pfmdr1* de-amplification results in increased MQ susceptibility. For example, in an artelinic acid (AL)-resistant line of *P. falciparum* (W2AL80) and clones originating from it, *pfmdr1* de-amplification resulted in partial reversal of resistance to AL and increased susceptibility to MQ, even in the absence of drug pressure^37^,^38^.

It is possible that both, reduced artemisinin susceptibility and MQ resistance during *Aotus* adaptation are due to increased growth rates. For example, treatment of neurosyphilis with the zoonotic malaria parasite *P. knowlesi* was eventually abandoned due to increased virulence of this species after multiple passages in humans^39^. We modeled PfC2A growth rates during *Aotus* adaptation and indeed demonstrated a significant increase in parasite multiplication rate from near 1 (passage I) to above 7 (passage X). It is therefore possible that the increased growth (i.e., parasite fitness) during serial passaging in *Aotus* resulted in altered drug susceptibility phenotypes. Similar observations have been made in the mouse malaria model, where increased fitness in *P. chabaudi* correlated with reduced susceptibility to artemisinin^30^.

In conclusion, we present a comprehensive study on *Aotus* adaptation of the multidrug resistant *P. falciparum* C2A strain. Combined phenotypic and molecular analysis of parasites across *Aotus* passages demonstrates that host adaptation has co-selected for reduced drug susceptibility in this non-human primate model. Our data also suggest that this model may be used for the evaluation of anti-malarial drugs against the recently detected Artemisinin resistant strains emerging from South-East Asia.

## Materials and Methods

### *Plasmodium falciparum* parasite strains

**Figure 5:**
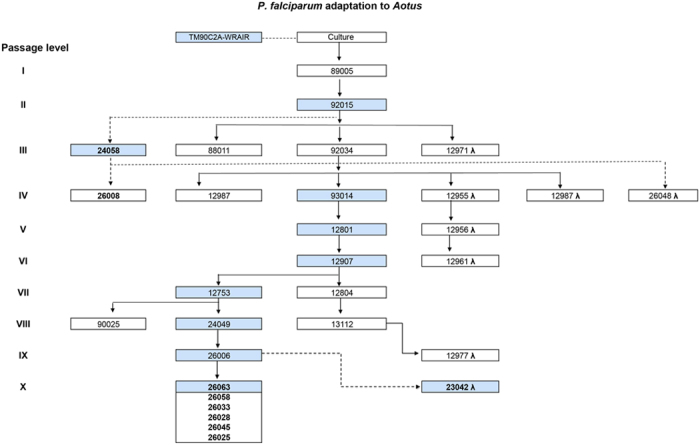
Genealogy *of Plasmodium. falciparum* C2A adaptation to *Aotus lemurinus lemurinus* monkeys. Blue highlighted squares indicate samples from which gDNA was extracted for genetic studies. *Aotus* monkey numbers in bold mark those that have been used in this study.

The original *P. falciparum* TM90C2A strain and subsequent passages in *Aotus* (Fig. 5)^15^, as well as *ex vivo* cultures thereof, were used in this study. In addition, the *Aotus* adapted strain *P. falciparum* FVO^34^,^40^ and *in vitro* adapted strains, D6, D10, CS2 and W2mef, were used as control parasites for these studies.

### Animals

Monkeys of the species *Aotus l. lemurinus* (Panamanian Owl monkey) Karyotype VIII & IX^41^ were used in this study. Animals were housed at Gorgas Memorial Institute of Health Studies (ICGES) in Panama, and cared and maintained as described^42^. Briefly, the animals were kept in stainless steel 4 unit quads cages (Lab Products Inc., Seaford, DE) with dimensions of 27 × 23.5 × 29.5 inches. Each cage was fitted with a 3⁄4-inch-diameter PVC pipe perch placed across 2/3 of the length of the cage and a 6-inch-diameter × 14.5 inches long PVC T pipe nest-box attached to the roof and back of the cage with cable zip ties. Cages were routinely cleaned and sterilized at 180° F at weekly intervals in a cage washing machine (Steris^®^, Erie, PA). During experimental infections the animals did not receive analgesics.

The experimental protocol was approved by the ICGES Institutional Laboratory Animal Care and Use Committee (CIUCAL) in accordance with procedures described in the “Guide for the Care and Use of Laboratory Animals,” 1996; protocol approval number 2006/02. All experiments described herein were performed in accordance with the approved guidelines.

### Drug efficacy studies in *Aotus* monkeys

To determine drug efficacy using the commonly used delivery route in humans, MQ and AS were applied orally alone or in combination. Six splenectomized male and female laboratory bred *Aotus* weighing between 758–829 g and one spleen intact control were inoculated intravenously (i.v.) in the saphenous vein with 5 × 10^6^ parasitized erythrocytes of the C2A strain from a donor monkey (passage IX). The animals were splenectomized ~30 days prior to inoculation as described^43^ (Fig. 5, Table 1). Parasite density was determined daily by Giemsa stained thick blood smears using the method described by Earle and Perez^44^, with 50 *μ*L of blood obtained with a lancet prick from the marginal ear vein. When parasitemia reached ~100,000 infected RBCs/μL, animals were assigned to one of the three treatment arms (groups) by weight and sex and treated as shown in Table S3. Drug doses were calculated on a milligram (mg) base per kilogram (kg) of body weight basis^45^. Oral administration of drugs was by gastric intubation with a 14 French red rubber catheter, while for i.v. administration a 25g butterfly needle was used. Response to treatment was categorized as no effect, parasite suppression without clearance, parasite clearance and recrudescence, or parasite clearance and cure. The day of parasite clearance was defined as the first of three consecutive days in which the thick blood films were parasite negative. The day of recrudescence was defined as the first of 3 consecutive days of positive thick blood films after a period of clearance. Parasite suppression was defined as a transient decrease in parasite density post-treatment without clearance^15^. Animals were considered cured if no recrudescence was observed after parasite clearance and 100 days of follow up with twice a week negative thick smears.

Before initiation of treatment, citrated whole blood was collected from each animal, plasma removed, and cryopreserved with Glycerolyte^®^ in liquid nitrogen and labeled passage level X for further *in vitro* IC_50_s determination, Ring Susceptibility Assay (RSA) and genotypic studies. Rescue treatment with MQ at 40mg/Kg orally once and AS at 20 mg/Kg i.v. for three days was triggered by any of the following: Hematocrit (HTO) below 50% of baseline, thrombocytopenia (<50 × 10^3^/μL) or signs of depression or anorexia as determined by the attending veterinarian. Two animals were also included in this study as historical references representing infections with C2A passages III (MN24058) and IV (MN26008) shown in Fig. 5 and Table S1.

### *In vitro* drug assays

*In vitro* IC_50_ values were obtained using the hypoxanthine incorporation assay^46^. IC_50_ cutoff values indicative of resistance were adopted from the literature as follows: >100 nM for CQ^21^,^22^,^23^,^47^, 30 nM for MQ^21^,^22^,^47^, 20 nM for AS^21^,^23^, and 12 nM for DHA^21^. Resistance cutoff points for QHS and ATV were not available from the literature. *Ex vivo* parasite passages were cultured for up to 8 serial passages in RPMI media containing 25% human AB + serum and 4% packed red blood cells. Thin smears were stained with Giemsa for morphological studies and detection of gametocytes. *In vitro* ring stage 0–3 hours survival assays (RSA) were carried out as described^24^ and the threshold for resistance was established at 1% survival.

### Genetic analysis of parasite strains

#### Molecular barcode and drug resistance genotyping

Genomic DNA was obtained from blood samples using phenol extraction-ethanol precipitation protocol for purification and concentration of DNA as described^48^. DNA concentrations of the samples were measured using a NanoDrop® spectrophotometer ND1000. Extracted DNA was pre-amplified and genotyped across 24 genomic loci with both high-resolution melting (HRM) and TaqMan technologies (Life Technologies, Grand Island, NY) as previously described^25^,^49^. Samples were diluted 1:20 and 5 μL of each pre-amplified product used for the molecular barcode. TaqMan barcoding assays were run on a ViiA system (Applied Biosystems) and genotypes called using the pre-installed analysis software. To confirm haplotypes and determine drug resistance genotypes of the C2A *Aotus* passages, we used a high resolution melting (HRM) analysis as described^50^.

#### *Pfmdr1* copy number variation (CNV)

To determine CNV in the *pfmdr1* gene across C2A *Aotus*-adapted passages, gDNA was extracted and used in a qPCR assay, as described^13^. *Pfmdr1* fold changes between C2A passage levels III-X and reference strain FVO were calculated as described^51^. Fold change results were rounded to the next significant integer for the purpose of determining copy number. Primer sequences are included in Table S2.

#### Gene sequencing

To determine single nucleotide polymorphisms (SNPs) in the *K13* propeller domain, PCR sequencing was carried out. The full ORF of K13 was PCR amplified using Phusion HF DNA Polymerase kit and primers 1F and 1R. The resulting PCR product of ~2.2kb was purified by gel extraction (QIAquick Gel Extraction Kit, Qiagen) and sequenced at Genewiz using primers 1F, 2F, 3F, 1R, 2R and 3R. Primer sequences are included in Table S2.

#### Chromosomal deletions

To determine whether common genomic deletions in the subtelomeric regions of either chromosome 2 or 9 occurred during C2A passages in *Aotus*, several loci were analyzed by PCR. For this purpose genomic DNA was extracted using Qiagen™ DNA kit across *P. falciparum* C2A *Aotus*-adapted passages, as well as *P. falciparum* reference strains FVO, D10 and 3D7. PCR amplification of genes *PF3D7_0201500* (*PFB0075c*) and *PF3D7_0202000* (*PFB0100c*) on chromosome 2, as well as *PF3D7_0935400* (*PFI1710w*)*, PF3D7_0936300* (*PFI1755c*), *PF3D7_0936800* (*PFI1780w*) on chromosome 9 were carried out in a BioRad™ PCR machine using the following amplification program: 95^o^ Celsius (C) × 5 minutes, 95 °C × 30 minutes, 51.4 °C × 30 minutes, 61.0 °C × 3 minutes for 35 cycles, plus 72 °C × 10 minutes. Amplicons were loaded onto a 1% agarose gel and subjected to electrophoresis at 150 Volts for 30 minutes, stained with ethidium bromide and photo-documentation done with a UV light reader. Primer sequences are included in Table S2; primers for *KAHRP* (*PFB0100c*) amplification were published previously^52^.

### Statistical analysis

Prism 5 Graph Pad^®^ Software was used to plot and calculate the mean and standard error of the mean (SEM), and the JMP® Pro 10.0.0 statistical program (SAS Institute Inc, Middleton, MA) was used for the Mann-Whitney U significance test between unpaired IC_50_ values for TM90C2A and passage levels II and X. Matlab R2013a was used to plot parasitemia curves and calculate parasite multiplication rate (PMR). PMR is modeled via polynomial regression using the polynomial degree corresponding to the lowest Akaike Information Criteria.

## Additional Information

**How to cite this article**: Obaldía, N. *et al.* Altered drug susceptibility during host adaptation of a *Plasmodium falciparum* strain in a non-human primate model. *Sci. Rep.* **6**, 21216; doi: 10.1038/srep21216 (2016).

## Supplementary Material

Supplementary Information

## Figures and Tables

**Table 1:** Parasitological treatment responses of *Aotus* monkeys infected with a *Plasmodium falciparum* C2A clone.

Group	ID	*Aotus*	Weight g.	Parasitemia	Day of peak	Group	Regimen mg/Kg	Route	Treatment	Day of treatment	Day rescue	Results primary
		Sex		Peak 103 × μL					Duration days			
1	26058	2	829	104.60	11	MQ	40	p.o.	1	11	14	Failed
1	26033	1	758	272.74	9	MQ	40	p.o.	1	9	5	Failed
2	22028	2	764	449.73	9	AS	33	p.o.	3	9	10	Failed
2	26045	1	789	110.58	11	AS	33	p.o.	3	11	8	Failed
3	26063	1	782	177.84	9	AS + MQ	33 + 40	p.o.	3 + 1	9	16	Failed
3	26025	2	785	106.02	10	AS + MQ	33 + 40	p.o.	3 + 1	10	None	Failed
4	23042 λ	1	724	0.340	16	AS + MQ	33 + 40	p.o.	3 + 1	16	None	Cured

Male = 1; Female = 2.

MQ = mefloquine; AS = artesunate.

λ = spleen intact.

p.o. = *per os.*
