# Supplementary material for: Altered drug susceptibility during host adaptation of a Plasmodium falciparum strain in a non-human primate model
Source: Sci Rep. 2016 Feb 16;6:21216. doi: 10.1038/srep21216 (PMC4754742; doi:10.1038/srep21216)
Supplement: Supplementary Information [file srep21216-s1.pdf]

TABLE S1. Lineage, parasitological, treatment, genotyping and antimalarial resistance profile of *Plasmodium falciparum C2A in Aotus monkeys*

Passage Level	ID	Inoculation Date	Donor	Inoculum Preservative description	Inoculation route	Inoculum Size	Day of Patency	Peak Asexual Parasitemia x uL x 10^3	Day of Peak	Day of primary Treatment	Primary treatment administration route	Day of Clearance	Days to clear from end of primary treatment	Primary treatment disposition	Day of Recrudesce	Day of Retreatment	Final Disposition	Treatment regimen (mg per kg/days;Day PI)	Production of Gametocytes Yes or No	Gametocyte day of patency	Gametocyte Peak x 10^3	Gametocyte PI Day of Peak	Gametocyte day of clearance	Gametocyte day of recrudescence
	TM90C2A-WRAIR																							
I	89005	12/14/98		Culture	IP	nd	73	10.50	84			90					self-cured	None	Yes	77	0.01	77	106	
II	92015	2/26/99	89005	Citrate	IV	>10	2	20.12	22	73	Oral	93	20	cured			cured	MQ (40/3;73)	Yes	6	260	82	7, 22, 33, 93	16, 24, 74
III	92034	5/7/99	92015	Citrate	IV	18120	1	85.12	11	11	Oral	55	43	failed	95		self-cured	MQ (20/1;11)	Yes	5	>0.01	14	7, 43	12,
III	24058	11/24/09	92015	Glycerol-EDTA	IP	42280	19	56.43	27	27	Oral/IV	31	2	cured			cured	MQ (40/1;27), AS_IV (30/3;27)	Yes	27	>0.01	29	30	
III	24058	11/24/09	92015	Glycerol-EDTA	IP	42280																		
III	24058	11/24/09	92015	Glycerol-EDTA	IP	42280																		
IV	93014	8/12/99	92034	Citrate	IV	>10	3	121.50	8	8		41	30	failed		22, 42	died	ART (8/3;8)(16/3;22), QN (20/2;43)	Yes	15	0.01	15	19, 21, 34, 37	20, 27, 36
IV	26008	12/21/09	24058	RPMI	IV	5.0 x 10^6	1	50.27	8	9	Oral/IV	13	2	cured				MQ (40/1;9), AS_IV (33/3;9)	Yes	9	>0.01	9	11	
IV	26048λ	12/21/09	24058	RPMI	IV	5.0 x 10^6	1	0.35	4			8					self-cured	None	No					
V	12801	5/2/02	93014	Glycerol-EDTA	IP	121500	7	113.52	25	26	Oral					33, 46, 48	died	MQ (20/1;26), QN (20/5;33), QN (20/2;46), PS26 (40/3;48)	No					
VI	12907	5/24/02	12801	Citrate	IV	4860	2	121.50	11	14	Oral	39	20	failed		26, 38,	cured	QN (20/5;14), PS26 (20/3;26), MQ (40/1;38)	No					
VII	12804	6/7/02	12907	Citrate	IV	23500	1	72.42	6	11	Oral					12		QN (20/1;11), PS26 (10/3;12)	No					
VIII	13112	6/18/02	12804	Citrate	IV	27750	1	87.75	6	7	Oral			failed		13	died	WR283178(40/3;7), MQ (40/1;13)	No					
VII	12753	7/13/07	12907	Glycerolate	IV	121510	15	6.59	21	21	Oral	28	7	failed	70	26, 81	cured	MQ (40/1;21), AS_IV (20/3;26), MQ (40/1;81) AS (8/3;81)	No					
VIII	90025	8/3/07	12753	Citrate	IV	6590	2	1.13	7	60	Oral	17		cured			cured	MQ (40/1;60), AS(8/3;60)	No					
VIII	24049	4/17/08	12753	Glycerolate	IV	6590	4	266.76	12	12	IV	24	10	failed	45	21, 69	cured	AS_IV (20/3;12), MAL (25/3;21), AS_IV (20/3;69), MQ (40/1;69)	No					
IX	26006	4/7/09	24049	Glycerolate	IV	266,760		115.25	7	8	Oral/IV	19	9	failed	14	17	cured	MQ (40/1;8), AS_IV (20/3;8), MQ (40/1;17), AS_IV (20/3;17)	No					
X	26058	4/15/09	26006	RPMI	IV	5.0 x 10^6	2	104.31	11	11	Oral	28	17	failed	20	27	cured	MQ(40/1;11), AS_IV(20/3;27), MQ(40/2;29)	No					
X	26033	4/15/09	26006	RPMI	IV	5.0 x 10^6	2	272.74	9	9	Oral	23	14	failed		16	cured	MQ(40/1;9), AS(33/3;16)	No					
X	26028	4/15/09	26006	RPMI	IV	5.0 x 10^6	1	449.73	9	9	Oral	23	12	failed		21	cured	AS(33/3;9), AS_IV(20/3;21), MQ(40/1;23)	No					
X	26045	4/15/09	26006	RPMI	IV	5.0 x 10^6	2	110.58	11	11	Oral	23	12	failed		21	cured	AS(33/3;11), AS_IV(20/3;21), MQ (40/1;23)	No					
X	26063	4/15/09	26006	RPMI	IV	5.0 x 10^6	2	177.84	9	9	Oral	28	17	failed		27	cured	MQ (40/1;9), AS (33/3;9), AS_IV (20/3;27), MQ (40/1;29)	No					
X	26025	4/15/09	26006	RPMI	IV	5.0 x 10^6	2	106.02	10	10	Oral	24	12	failed	20		cured	MQ(40/1;10), AS(33/3;10)	No					
X	23042λ	4/15/09	26006	RPMI	IV	5.0 x 10^6	2	0.34	17	16	Oral	18	-1	cured			cured	AS(33/3;16), MQ(40/1;18)	No					
MIN							1	0.34	4	7		8	-1		14					5		9		
Q1							1	17.715	7.75	9		19	7.5		18.5					5.75		12.75		
Median							2	96.03	10.5	11		24	12		32.5					12		22		
Q3							3.5	121.5	18	19.75		39	19.25		76.25					39.5		78.25		
MAX							73	449.73	84	73		93	43		95					77		82		

λ = Spleen intact
* = Repeat
** = Pfldh

Primer sequences

MDR1-T1F 5'-TATGCATTTGTGGGAGAATCAG-3'

MDR1-T1R 5'-CTCCTTCGGTTGGATCATAAAG-3'

LDH-T1F 5'-AGGACAATATGGACACTCCGAT-3' and

LDH-T1R 5'-TTTCAGCTATGGCTTCATCAAA-3'

1F: 5'-ATGGAAGGAGAAAAAGTAAAAACAAAAGCAAATAG-3'

2F: 5'-GGTAGGTGATTTAAGAATTACATTTATTAATTGGT-3'; 3F: 5'-CATTCCCATTAGTATTTTGTATAGGTG-3'

1R: 5'-TTATATATTTGCTATTAAAACGGAGTGACCAAATCTG-3'

1R': 5'-TTA TAT ATT TGC TAT TAA AAC GGA GTG-3'

2R: 5'AGCCTTATAATCATAGTTATTACCACCAAAAACG-3'

3R: 5'-TGTTGGTATTCATAATTGATGGAGAATTC-3'

PFB0075c-F 5'-GGGCGGCCGCATGAAAATATGAGAAGTTTAAAG-3'

PFB0075c-R 5'-CCGCTAGCACTAGTGAATATAATAAAAATTACGATGGAGAA-3'

PFI1710w-F 5'-CCTGAACACTTGAAAATGGAAA-3'

PFI1710w-R 5'-AAACGAAAAGATTGAAATGGA-3'

PFI1755c-F 5'-ACCTGATCCTAGTGTACCCTCTCT-3'

PFI1755c-R 5'-GAAGAACTTGTACTTGGTTTAGCCG-3'

PFI1780w-F 5'-AAAACTAGTATGTTTTTATGTAGGTTATGAG-3'

PFI1780w-R 5'-AAACTTAAGCTCTCAGATTCATGTACATAAC-3'

Table S3. Average parasite day of clearance of *Plasmodium falciparum* infections in splenectomized or spleen intact Aotus monkeys after treatment with mefloquine and artesunate alone or in combination

	Aotus infection groups				
P. falciparum strain	C2A	C2A	C2A	C2A	C2A
Passage level	III-IV	X	X	X	X
Splenectomized	*y*	*y*	*y*	*y*	*n*
	Treatment regimens				
Drug	AS / MQ	MQ	AS	AS / MQ	AS / MQ
mg/Kg x (days)	33 (3) / 40 (1)	40 (1)	33 (3)	33 (3) / 40 (1)	33 (3) / 40 (1)
Route	*i.v. / po*	*po*	*po*	*po*	*po*
	Treatment outcome (individual Aotus day of clearance*)				
	5	15**	15**	15	3
	5	17**	13**	20**	-
	-	-	-	-	-
Average	5	16	14	17.5	3

* = day after inititation of treatment — *i.v.* = intravenously — *po* = *per os* (orally)

** = did not clear with primary treatment — *nd* = not determine — *y* = yes; *n* = no

AS = artesunate — MQ = mefloquine

Genomic analysis of chromosomes 2 and 9

Chromosome 2

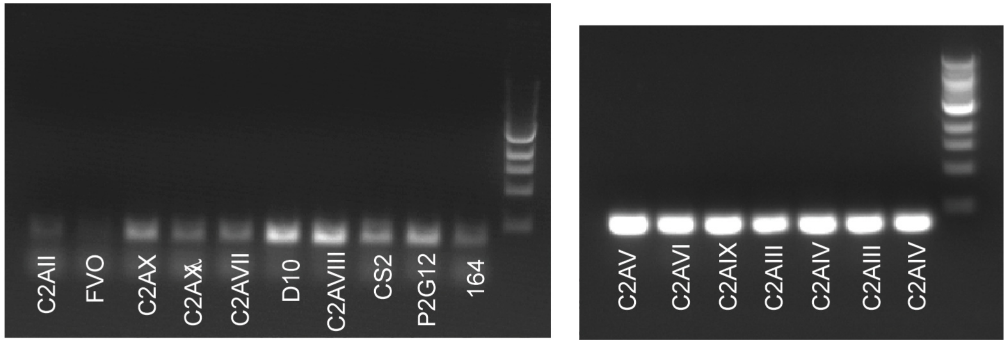

PFB0075c (Hyp9)

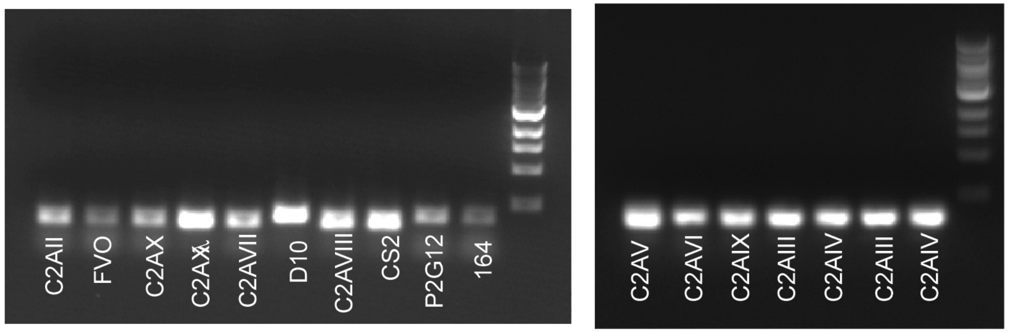

PFB0100c (KAHRP)

Chromosome 9

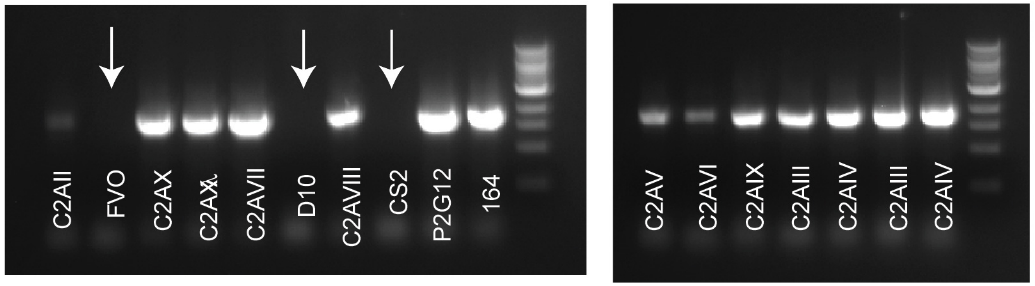

PFI1710w (GDV1)

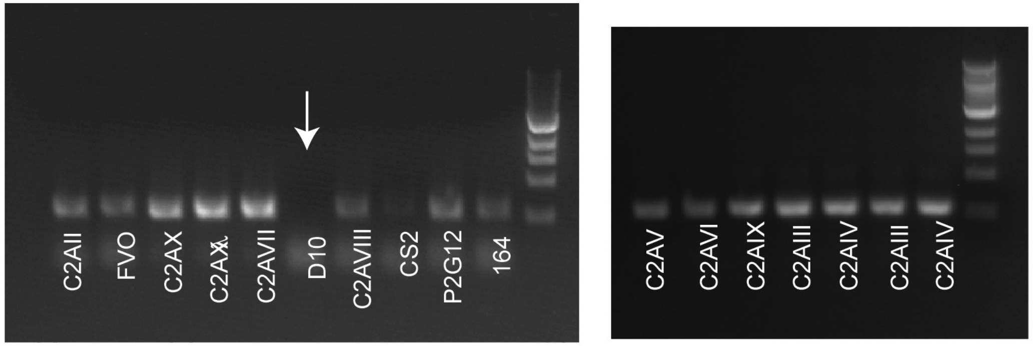

PFI1755c (Rex3)

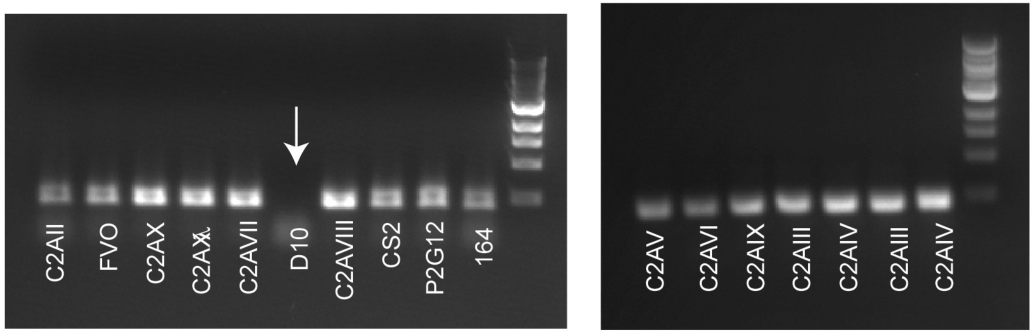

PFI1780w (PHISTc)
